# Effects of empagliflozin on the expression of kisspeptin gene and reproductive system function in streptozotocin-induced diabetic male rats

**DOI:** 10.3389/fendo.2022.1059942

**Published:** 2022-11-21

**Authors:** Parisa Dana, Nasim Hayati Roodbari, Parichehreh Yaghmaei, Zahra Hajebrahimi

**Affiliations:** ^1^ Department of Biology, Science and Research Branch, Islamic Azad University, Tehran, Iran; ^2^ A&S Research Institute, Ministry of Science Research and Technology, Tehran, Iran

**Keywords:** empagliflozin, kisspeptin gene, reproduction, streptozotocin, diabetes mellitus, Wistar rat

## Abstract

One of the main health concerns of diabetes is testicular dysfunction and impairment of reproductive function and sperm quality which can cause male infertility. kisspeptin is a hypothalamic neuropeptide hormone that is involved in the regulation of energy metabolism, gonadotrophin-releasing hormone (GnRH), and reproductive function. In the present study, the therapeutic effects of empagliflozin (sodium-glucose co-transporter 2 inhibitors) on kisspeptin expression along with reproductive function were investigated in diabetic male Wistar rats. Diabetes was induced by a single dose injection of 60 mg/kg streptozotocin. Empagliflozin in doses of 10 and 25 mg/kg body weight was used for 8 weeks. Serum samples, testis, epididymis, and pancreas tissues were collected at the end of the experiments. Lipid profiles, oxidative stress markers, blood hormones, expression of kisspeptin along with pathological alterations of the testis were assayed using real-time PCR, biochemical, and histological technics. Data have shown that empagliflozin improved hyperglycemia, reproductive impairment, oxidative stress condition, and histopathological alterations of pancreatic and testis tissues in diabetic animals. It improved the serum levels of sex hormones, insulin, leptin, and the expression of kisspeptin in the testes tissues. Spermatogenesis is also improved in treated animals. Data indicated that the administration of empagliflozin can ameliorate symptoms of diabetes. It probably has promising antidiabetic potential and may improve the male infertility of diabetic subjects. To our knowledge, this is the first experimental evidence for the potential impact of empagliflozin on kisspeptin expression in diabetic male rats.

## Introduction

Diabetes mellitus is a metabolic disorder that is characterized by insulin resistance, hyperglycemia, dyslipidemia, and hypertension (1–4). Oxidative stress, inflammation, and obesity which subsequently can lead to the production of oxidative stress and inflammation have a critical role in the development of diabetes (1–7).

In most countries of the world, the prevalence of diabetes is higher in middle-aged men than in middle-aged women (8). Modulation of energy and regulation of glucose metabolism is different in men and women, and gender affects the susceptibility to diabetes (9).

The secretion of steroid hormones after puberty plays a critical role in gender-related differences in the incidence of diabetes and susceptibility. Sex hormones are the main biological regulators of the islets of Langerhans. Studies suggested that estrogens can stimulate the biosynthesis and secretion of insulin and can regulate the metabolism of glucose (10, 11). It has protective roles on the islets of Langerhans and is involved in the proliferation, survival, maintenance, and function of beta cells (11–13). Also, it has been reported that estrogens can prevent metabolic damage including oxidative stress and fat toxicity that leads to beta-cell apoptosis (11). Evidence suggests that the amount of beta cells in the islets of Langerhans is 6% higher in females than males (9, 14).

One of the main health concerns of diabetes is a disturbance in the production of testosterone and therefore sperm quality which can cause male infertility. Glucose metabolism is required for the maintenance of spermatogenesis. Many studies have demonstrated the adverse effects of diabetes on male fertility such as a decrease in semen volume, sperm concentration, sperm motility, and seminal tubes diameter; an increase in degenerative germ cells, sperms and Sertoli cells with abnormal morphology, sperm DNA damage; the accumulation of lipid droplets in Lyding cells; and erectile dysfunction (15–17). Studies by Zhang et al. (2015) was shown that obesity and inflammation, two key attributes in the development of diabetes, could impair the quality of sperm in male mice and decrease the amounts of testosterone in blood serum (2). These may be due to an increase in the cholesterol rate of the sperm membrane and an increase in oxidative stress in obese individuals (3–7)

Kisspeptin (KP) is a hypothalamic neuropeptide hormone that regulates reproductive function. It is encoded by the *KISS1* gene in humans that binds to the G-protein coupled receptor (GPR54 or KISS1R) (18). KP is involved in the stimulatory regulation of the gonadotrophin-releasing hormone (GnRH), puberty onset, and fertility. GnRH stimulates gonadotrophins including luteinizing hormone (LH), and follicle-stimulating hormone (FSH)) and downstream sex hormones such as testosterone and estradiol (19–21). The expression of KP was shown in a variety of tissues including the neurons of the hypothalamus, adrenal gland, pancreas, liver, and adipose tissue (21–23). KISS1R is expressed in the acrosome region of spermatids, spermatozoa, and Leydig cells (19). Therefore, the secretion of KP and activation of its receptor signal the release of gonadotropin, which modulates the production of sex hormones in both males and females (19–23). Also, studies in primates, rodents, amphibians, and fish have demonstrated an important function of kisspeptin in germ cell progression, modulation of sperm function, and testicular steroidogenesis (24).

Apart from the regulatory function of KP in the reproductive process, recent studies suggested its role in the regulation of metabolism and energy balance. In fact, alteration in the expression of KP was reported in metabolic disorders such as diabetes and obesity (25). KISS1R regulates the secretion of insulin from beta cells of the pancreas (26). It has been demonstrated that the levels of KP decreased in the pancreas of male rats with diabetes (21). In addition, both metabolic state and energy balance are important for reproduction, too (27).

Empagliflozin is in a class of anti-diabetic drugs called sodium-glucose co-transporter 2 (SGLT2) inhibitors with anti-hyperglycemic activity. It is used along with other medications, to lower blood sugar levels by suppressing the reabsorption of glucose in the proximal tubule of kidneys upon inhibition of SGLT2 (28, 29). Evidence indicates that it has anti-oxidant and anti-inflammatory activity, too (29). In the present study, we investigated the effect of Empagliflozin on the expression of the kisspeptin gene and reproduction parameters in male rats with diabetes.

## Materials and methods

### Animals and experimental design

For this study, a total number of sixty adult male Wistar rats weighing 200 ± 20 g were used. The animals were provided by the Laboratory Animal Sciences of Pasteur Institute, Karaj, Iran. Before initiating the experiments, the rats were adapted to the environmental condition for two weeks in the Razi laboratory animal of Islamic Azad University, Science and Research Branch, Tehran, Iran. The rats were housed 4 per cage under standard laboratory conditions in animal rooms with controlled temperature (25 ± 1°C), humidity condition (50 ± 15%), 12-h light/dark cycle (lights out between 6:00 PM and 6:00 AM), and well air ventilation (8-15 times per hour). Throughout the experiment, rats were free to eat and drink. All experimental protocols were conducted in accordance with the guidelines for the Care and Use of Laboratory Animals (Committee for the update of the guide for the care and use of laboratory animals, 1996). All protocols were approved by the Animal Care and Use Committee of Islamic Azad University, Science and Research Branch (permit number: IR.IAU.SRB.REC.1399.184).

### Induction of diabetes

Diabetes was induced in rats by intraperitoneal injection of 60 mg/kg streptozotocin (STZ; Sigma-Aldrich Chemie GmbH - Schnelldorf, Germany) dissolved in 0.01 M sodium citrate buffer at pH 4.5. One week following the STZ injection, blood samples were taken from the tail vein and glucose level values were measured using a glucometer (Cera pet, South Korea). Rats with blood glucose levels above 18 nmol/L were considered manifestly diabetic (30). The dosages of the STZ in the present study were chosen based on earlier studies (31).

### Animals grouping

Animals were randomly categorized into five groups (n=12) as follows: 1- control group (C) that were fed with a standard diet and did not receive any treatment; 2- diabetic group (D) with an intraperitoneal injection of 60 mg/kg streptozotocin; 3- sham operation group (sham): diabetic rats that received distilled water (5 CC per day) for 8 weeks; 4- Empagliflozin-10 group (E10): diabetic rats that received Empagliflozin (10 mg/kg/day) for 8 weeks; 5- Empagliflozin-25 group (E25): diabetic rats that received Empagliflozin (25 mg/kg/day) for 8 weeks. The supplemented empagliflozin or distilled water was given by oral gavage.

Thereafter, the animals were kept under standard conditions and monitored for 56 days. The dosages and duration of the treatment in the present study were chosen based on earlier studies (32–34). The body weights of rats were measured at the beginning and at the end of the experimental period.

### Analysis of hormonal and biochemical parameters

Food and water consumption (in terms of g and ml, respectively) were measured using metabolic cages (35). For measurement of urine glucose, animals were placed in metabolic cages fasting for 12 hours and urine samples were obtained. Urine glucose concentrations were measured using a commercial urine glucose assay kit (Beijing Applygen Technologies Inc., China) according to the instructions of the manufacturer (35).

At the end of the eighth week, animals were fasted overnight, anesthetized by injection of 80 mg/kg of ketamine and 10 mg/kg of xylazine, and blood samples were taken from the heart. Blood samples were left at room temperature for 2 hours in order to get clotted. Then, serum samples were collected through centrifugation at 3000×g for 10 minutes and stored at -20°C until biochemical analysis (36).

Analysis of biochemical parameters such as glucose, total cholesterol (TC), triglycerides (TG), high-density lipoprotein (HDL), and low-density lipoprotein (LDL) was measured by commercially available animal spectrophotometric assay kits (Pars Azmoon Company, Karaj, Iran) based on the manufacturer’s recommendations.

The activity of MDA (Malondialdehyde) and superoxide dismutase (SOD) were measured by Nasdox and NalondicELISA kits, respectively (Navandsalamat Co., Urumia, Iran) based on the manufacturer’s instructions. The activity of catalase (CAT) was measured using commercial ELISA kits (Zellbio GmbH, Ulm, Germany) according to the instructions of the manufacturer.

Serum levels of insulin, testosterone, luteinizing hormone (LH) and follicle-stimulating hormone (FSH) were measured using rat/mouse ELISA test kits (Cosmo Bio Co. Ltd. Japan) based on the manufacturer’s instructions.

Serum levels of adiponectin and leptin were measured using ELISA test kits (Biovendor Company, Czech) based on the recommendations of the manufacturer.

### Testicle, epididymis, and pancreatic tissue collection

At the end of the experiment period, animals were sacrificed and testes, epididymis, and pancreatic tissues were collected. Testes were washed, weighed, and their dimensions were measured. The left epididymis was collected for sperm motility and sperm morphology evaluation. The left testis was removed, froze in liquid nitrogen, and stored at -70 °C for further molecular analysis. The right testis was fixed in Bouin’s solution for further histological evaluation. Pancreatic tissues were removed, rinsed in chilled normal saline, and fixed in formalin (10%) for further histological evaluation.

### Gonadosomatic index and sperm quality analysis

To evaluate sperm motility, the caudal region of the epididymis was isolated and a cut was made on the head of the epididymis. Then, the fragments were placed into the phosphate-buffered saline (PBS) and incubated at 37˚C for 10 minutes. The motility of sperms was determined by a standard hemocytometer method using Neubauer slide (37) and evaluated under a light microscope (x100) in accordance with the WHO standard method for manual examination of sperm motility (38). The motility (%) was presented using the number of motile sperms over the total number of sperms (motile+nonmotile sperm).

For the evaluation of sperm morphology, the sperm smears were prepared on the histological slides and allowed to air-dry. After drying, the slides were observed under the light microscope at x400 magnification. From each rat, a total of 100 sperms were observed and evaluated defects for the head and tail. The percentage of sperms with abnormal heads or tails and the percentage of normal sperms were expressed.

Gonadosomatic index (GSI)was measured using the weight of the body and testis from the following formula: [Testes weight (g)/Bodyweight (g)] *100 (39). Testicular width and length were estimated with the aid of digital Vernier calipers as described by Pahizkar et al. (2014) (40). Testicular volume was measured using the water displacement method as described previously by Benjamin and Heideman (2002) (41).

### Histological procedures

After the fixing of the testis, epididymis, and pancreatic tissues, standard processing of dehydration in ethanol and clearing in xylene was performed. Following the embedding in paraffin, the paraffin blocks were subjected to serial sectioning of 5μm thickness by a rotary microtome and then processed in alcohol-xylene series and mounted on glass slides. Testes and epididymis samples were stained with hematoxylin and eosin (H & E) according to standard staining protocols. Histopathological assessment of the stained tissues was performed under a light microscope and analyzed with Image J software (NIH). The diameter of the seminiferous tubules, and the numbers of spermatogonia, spermatid, spermatocyte, and Sertoli cells were evaluated in the testes’ tissues. In pancreatic tissues, the percent of beta cells, the number of pancreatic islets per square centimeter, and the average area of pancreatic islets were evaluated (42).

### Molecular investigations

Total RNA was extracted from testes samples using RNX plus solution (Cinnagen, Iran) based on the manufacturer’s instruction and stored at −80°C until further examination. The concentration of RNA was identified using the NanoDropTM 2000 Spectrophotometer (Thermo Fisher Scientific, USA) and the purity of them was quantified by optical density at 260 and 280 nm. The verification of the integrity of the RNAs was identified using electrophoresis on 1% agarose gel. In order to remove genomic DNA, extracted RNA was treated with RNAase‐free DNAaseI (Thermo Fisher Scientific, MA). cDNA was synthesized using the Easy cDNA Synthesis Kit (Parstous, Iran) based on the manufacturer’s instructions. Quantitative real-time PCR was performed to assay the expression of *kisspeptin* for each sample relative to *β-actin* as the internal control gene. PCR amplification was done using 2X SYBR^®^ Green Real-Time PCR Master Mix (Parstous, Iran) following the manufacturer’s instructions. The RNAs were assayed in duplicate reactions using a StepOnePlus™ Real-Time PCR System (Applied Biosystems, USA) with gene primers listed as follows: Kisspeptin (accession number: NM-181692, product size: 110 bp): 5′-TGCTGCTTCTCCTCTGTGTGG-3′ (forward primer), 5′-ATTAACGAGTTCCTGGGGTCC-3′ (reverse primer), β-actin (accession number: NM-031144, product size: 122 bp): 5′-TCTATCCTGGCCTCACTGTC-3′ (forward primer); 5′-AACGCAGCTCAGTAACAGTCC-3′ (reverse primer). Primers were manufactured by Pishgam (Pishgam Biotech Co., Iran). The following thermal profile was set: 94°C for 10 minutes as hold time; 40 cycles of denaturation at 95°C for 15 seconds, annealing at 62°C (for Kisspeptin) and 58°C (for β-actin) for 30 seconds, and extension at 72°C for 45 seconds; and final extension at 72°C for 5 minutes. Continuous melt curve stages included the first step of 95°C/15 seconds, step 2 at 60°C for 1 minute, and the last step of 95°C/15 seconds. Relative expressions of Kisspeptin to β-actin mRNA were calculated using the 2^−ΔΔCt^ method. All reactions were evaluated in duplicate. To verify the specificity of PCR products, melt curves were obtained for both genes. Continuous melt curve stages were set as follows: the first step of 95°C for 15 seconds, step 2 at 60°C for 60 seconds, and step 3 at 95°C for 15 seconds.

### Statistical analysis

The data was analyzed using SPSS program version 22.0 (SPSS Inc, Chicago, IL, USA). All values are given as mean ± SEM. Kolmogorov–Smirnov test was used to determine the normal distribution of data. Normally distributed data were analyzed by One-Way Analysis of Variance (ANOVA) followed by Tukey’s post-hoc test. Not normally distributed data were analyzed by the Mann–Whitney test. In addition, a p-value of less than 0.05 was considered significant.

## Results

### Body and testis weights, food gain, and water intake

Injection of streptozotocin decreased body weights in diabetic rats. A significant difference was observed in the final values of the body weights when diabetes (D) and sham operation rats were compared with control (C) animals as represented in **Table 1** (P ≤ 0.001). Administration of empagliflozin caused a further decrease in body weights of empagliflozin-10 (E10) and empagliflozin-25 (E25) groups in comparison whit D and sham operation groups (P < 0.05).

There were significant changes in the weights of the testis in the STZ-treated groups (D and sham) when compared with the control group. STZ treatment decreased the weights of the testis in the D and sham groups, while administration of empagliflozin compensated for the effects of streptozotocin on testis weights in the E10 and E25 groups as recorded in **Table 1**. STZ treatment had no effects on the dimensions of the testis and GSI in the D and sham operation groups (P ≥ 0.05).

**Table 1:** Physiological, biochemical, and histological findings of the diabetic rats and controls.

	control (C)	sham	Diabetes (D)	Empagliflozin (10mg/kg)	Empagliflozin (25mg/kg)
Consumed food (g/24h)	28.57±3.28	46.75±5.29 *	47.22±5.19 *	50.98±3.31 **	53.99±4.90 **
Consumed water (ml/24h)	19.42±0.95	64.28±3.39 ***	68.20±4.30 ***	90.47±5.12 ***#+	114.75±10.04 ***###
Bodyweight (g)	300.16±8.28	184.42±10.11 ***+	175.54±11.28 ***	148.18±5.30 ***#	143.32±6.63 ***#
Urine glucose (mmoL/L)	2.68±0.73	45.06±3.38 ***+++	49.13±4.39 ***+++	75.66±2.71 ***###+	94.27±7.29 ***###
Glucose (nmol/L)	6.36±0.58	20.99±0.80 ***+++	21.76±0.79 ***+++	16.95±1.50 ***##++	12.22±0.78 ***###
Insulin (µIU/ml)	1.54±0.10	0.87±0.07 ***++	0.97±0.06 ***+	1.07±0.07 ***	1.28±0.07 #
Triglycerides (mg/dl)	70.38±4.96	95.21±4.82 *	95.15±6.49 *	103.20±6.33***	99.30±5.58 **
Cholesterol (mg/dl)	90.09±3.85	110.01±6.50 *	115.53±5.66 **	117.07±4.50 **	116.07±3.98 **
LDL (mg/dl)	34.60±2.61	74.33±4.30 ***	70.06±3.00 ***	78.77±3.03 ***	75.74±4.93 ***
HDL (mg/dl)	44.11±3.43	24.70±1.65 ***	21.01±0.98 ***+	25.13±1.66 ***	29.85±1.98 ***#
Adiponectin (ng/ml)	6.61±0.29	5.77±0.41	5.99±0.42	5.45±0.19	5.58±0.18
Leptin (ng/ml)	16.38±0.97	6.39±0.49 ***+	6.33±0.47 ***++	9.10±0.55 ***#	9.35±0.51 ***##
SOD (U/ml)	19.18±0.86	9.85±0.68 ***++	9.43±0.57 ***+++	14.15±0.78 ***###	13.94±0.79 ***###
Catalase (U/ml)	16.83±0.44	6.80±0.53 ***+++	7.61±0.49 ***+++	9.36±0.70 ***+++	12.88±0.67 ***###
MDA (μmol/l)	9.75±0.63	18.78±0.85 ***+	19.87±0.85 ***++	14.10±0.89 **###	15.41±0.90 ***##
FSH (IU/l)	4.21±0.23	1.39±0.12 ***	1.55±0.12 ***	1.35±0.14 ***	1.99±0.22 ***
LH (IU/l)	0.40±0.05	0.17±0.02 ***++	0.15±0.02 ***++	0.21±0.02 ***	0.33±0.04 ##
testosterone (ng/ml)	3.99±0.41	1.31±0.15 ***++	1.44±0.14 ***++	1.90±0.22 ***	2.85±0.34 *##
Spermatogonia (n)	63.63±4.63	35.13±4.22 **+	34.44±5.29 **+	47.19±4.18	52.88±3.20 #
Spermatocyte (n)	60.94±4.07	29.94±1.94 ***+	30.81±2.10 ***+	38.19±2.34 ***	41.00±1.72 ***#
Spermatid (n)	215.81±8.25	131.38±10.91 ***+	126.50±9.61 ***++	174.00±16.59	187.38±15.57 ##
Sertoli cells (n)	20.31±1.07	17.44±1.05	16.69±0.99	17.31±0.99	17.56±0.96
Diameter seminiferous (μm)	284.47±10.70	246.20±12.34 *	235.00±7.74 **	217.25±4.54 ***	223.63±7.30 ***
Testicular dimeter (mm)	10.39±0.74	9.52±0.57	9.72±0.73	9.95±0.66	9.71±0.57
Testicular length (mm)	22.33±0.75	21.48±0.65	20.15±0.63	20.74±0.69	20.71±0.73
Testicular volume (mm3)	3.90±0.25	3.79±0.19	3.47±0.19	3.90±0.23	3.57±0.21
Testicular weight (g)	1.58±0.09	1.05±0.15 *	1.09±0.13 *	1.21±0.12	1.14±0.10
GSI	0.54±0.04	0.60±0.09	0.55±0.05	0.82±0.08	0.79±0.06
Normal morphology sperm (n)	93.69±0.82	43.88±3.25 ***	49.44±3.07 ***	41.69±1.68 ***	46.50±2.18 ***
Abnormal sperm head (n)	4.69±0.71	28.50±1.68 ***	32.25±2.37 ***+	29.19±2.64 ***	24.88±1.14 ***#
Abnormal sperm tail (n)	2.00±0.30	15.75±0.78 ***+++	17.31±0.73 ***+	12.19±0.92 ***##+++	21.38±1.28 ***#
Sperm Motility (%)	77.88±0.75	37.50±0.55 ***++	39.75±0.55 ***+	42.94±0.56 ***	57.38±0.55 **##
β cells (%)	30.88±0.91	11.31±2.23 ***+++	12.69±2.03 ***++	20.00±1.86 **	23.44±2.13 ##
number of islets/sq.cm	71.06±0.84	44.88±1.57 ***++	45.38±1.49 ***++	50.75±1.81 ***	53.81±1.70 ***##
Islet area (µm^2^)	10648.81±81.51 ***	4531.06±147.92 ***+++	4435.00±177.93 ***+++	4826.13±288.19 ***++	6063.81± 264.56 ***###

Values are presented as mean ± SEM. (n=12). * Statistically different from the control rats, # statistically different from the diabetes rats, + statistically different from the E25 rats. *, #, and + = P ≤ 0.05; **, ##, and ++ = P < 0.01, ***, ###, and +++ = P < 0.001.

Food intake and water consumption increased significantly after the injection of streptozotocin (P < 0.05) in diabetic rats (**Table 1**). Administration of empagliflozin caused a further increase in water consumption in E10 and E25 groups when compared with D and sham groups, in a dose-dependent manner. Empagliflozin treatment had no effects on food intake in E10 and E25 groups in comparison to D and sham groups (P ≥ 0.05). Similar results were observed for levels of glucose in urine. The urine glucose values significantly increased in the D and sham groups in comparison to the control ones. Administration of empagliflozin caused a further increase in the urine glucose values in E10 and E25 groups in comparison to D and sham groups in a dose-dependent manner.

### Biochemical characteristics

The serum levels of glucose (Glu), triglyceride (TG), total cholesterol (TC), low-density lipoprotein (LDL), high-density lipoprotein (HDL), insulin, leptin, and adiponectin were summarized in **Table 1**. Injection of streptozotocin significantly increased the serum value of Glu, TC, TG, and LDL while decreasing the amount of HDL, insulin, and leptin in the D and sham groups when compared to the control group. STZ treatment had no effects on adiponectin levels (p>0.05). The administration of empagliflozin improved the amount of Glu, insulin, HDL, and leptin (Glu in E10 and E25 groups in a dose-dependent manner, insulin and leptin in E10 and E25 groups, and HDL in E25 group) in comparison to D and sham groups. There were no significant differences in serum values of TG, TC, and LDL in the E10 and E25 groups when compared with the D and sham groups (p>0.05).

The serum levels of LH, FSH, and testosterone are presented in **Table 1**. We observed that injection of streptozotocin decreased the serum level of FSH, LH, and testosterone in D and sham rats in comparison to the control animals. However, the levels of LH and testosterone improved following empagliflozin administration in the E25 group in comparison to the D and sham groups. Empagliflozin treatment had no effects on the FSH levels. Similar results were obtained for markers of oxidative stress (**Table 1**). We observed that injection of streptozotocin decreased the serum level of SOD and CAT, and increased the levels of MDA in diabetic and sham rats in comparison to control animals. However, their levels improved following the empagliflozin administration in comparison to the D and sham groups.

### Sperm quality analysis

**Figure 1:**
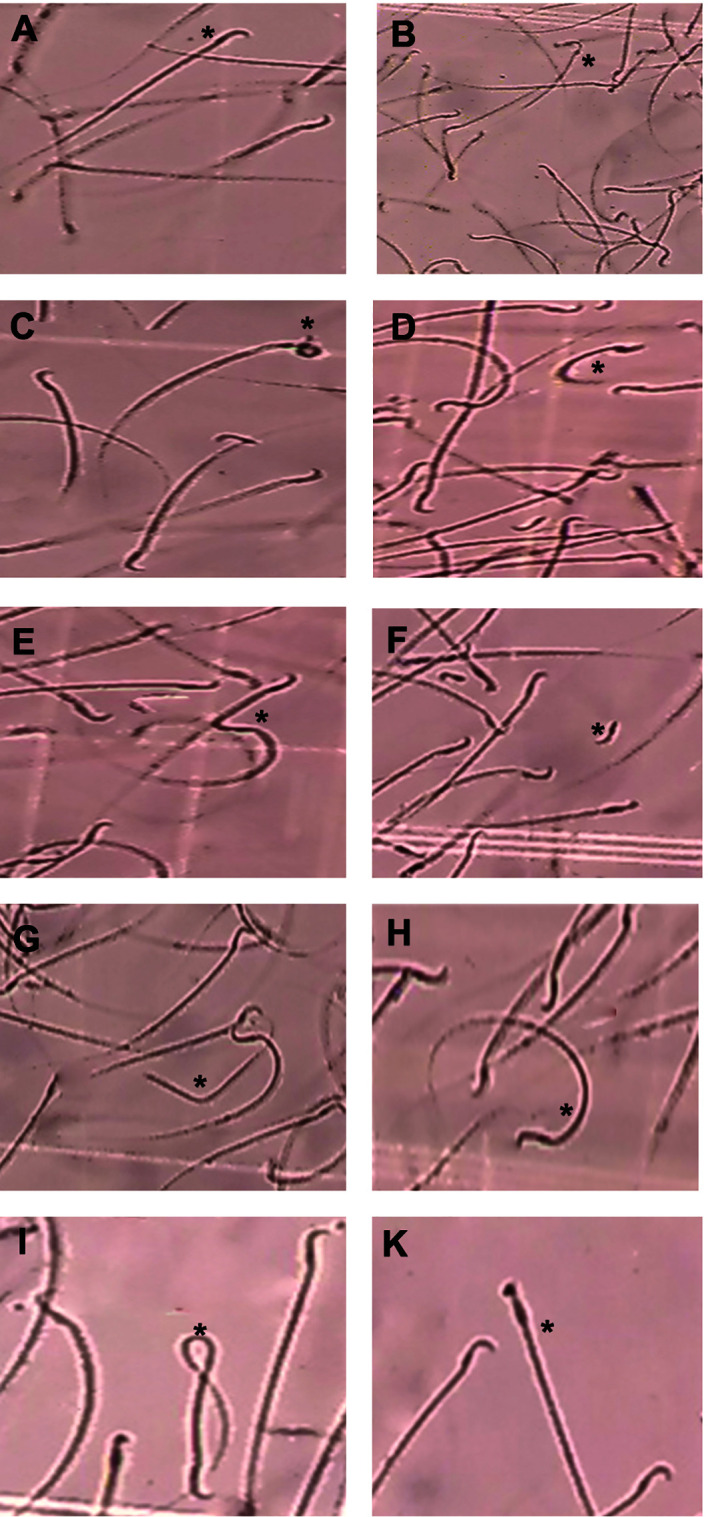
Microphotographs showing morphologically normal sperm and various sperm defects from the epididymis. **(A)** normal morphology, **(B)** Bent neck with cytoplasmic droplet, **(C)** head defects (large head), **(D)** tail defects (short and curve tail), **(E)** tail defects (broken tail), **(F)** detached head, **(G)** headless and broken tail sperm, **(H)** curve tail, **(I)** coiled tail, **(K)**: thick neck (magnification ×400).

Results of epididymal sperm analysis of all animal groups are represented in **Table 1**. The sperm motility analysis showed a marked decrease in sperm motility of animals from the D and sham groups compared with the control group. The morphological evaluation revealed that injection of streptozotocin markedly decreased the number of sperm with normal morphology in the D and sham groups compared with the control group. In contrast, markedly increased sperms with abnormal heads or tails were observed in STZ-treated groups (D and sham) in comparison to the control animals as recorded in **Table 1**. Empagliflozin administration improved sperm motility and the frequency of normal and abnormal sperms. Administration of empagliflozin significantly increased the number of normal sperms with normal motility. Also, the number of sperm with normal heads and tails significantly increased in the E25 group following the treatment with empagliflozin for 8 weeks. **Figure 1** shows the microphotographs of sperm morphology.

### Histopathological results

**Figure 2:**
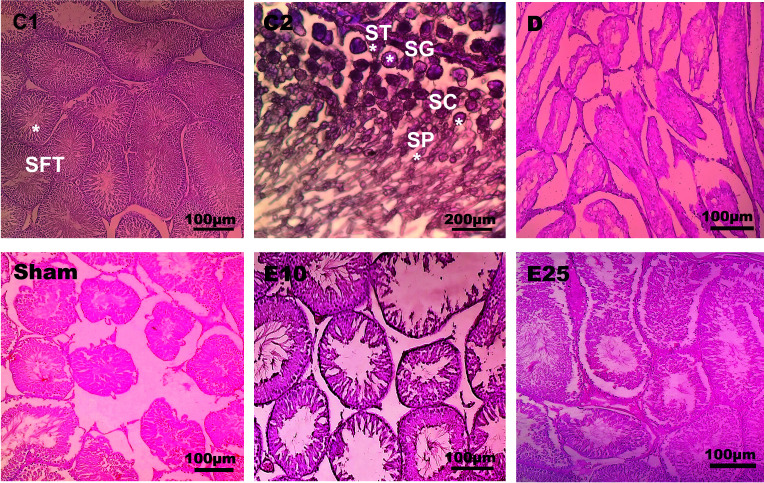
Testis cross-section. C1: control rats (magnification x400), C2: control rats (magnification x1000), D: diabetes group, sham: diabetic rats that received distilled water for 8 weeks, E10: empagliflozin-10 group (diabetic rats that received 10 mg/kg/day empagliflozin for 8 weeks, E25: empagliflozin-25 group (diabetic rats that received 25 mg/kg/day empagliflozin for 8 weeks. SFT: seminiferous tubules, SG: spermatogonia, ST: Sertoli cells, SC: spermatocyte, SP: spermatid. Magnification of D, sham, E10, and E25: x400.

**Figure 3:**
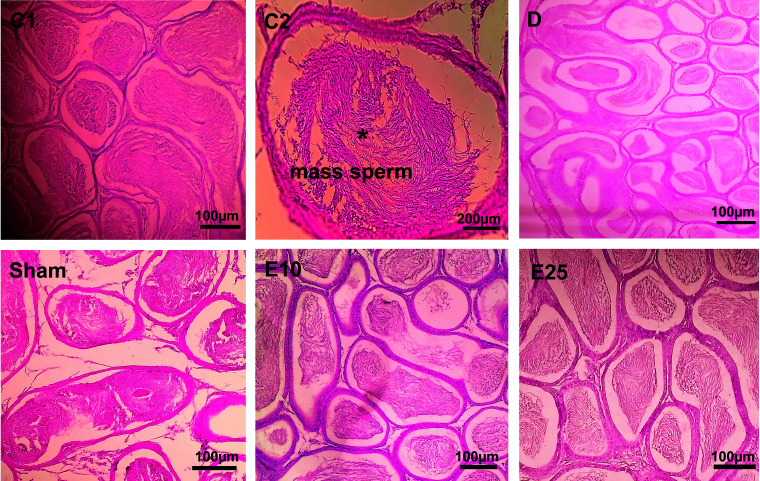
Epididymis cross-section. C1: control rats (magnification x400), C2: control rats (magnification x1000), D: diabetes group, sham: diabetic rats that received distilled water for 8 weeks, E10: empagliflozin-10 group (diabetic rats that received 10 mg/kg/day empagliflozin for 8 weeks, E25: empagliflozin-25 group (diabetic rats that received 25 mg/kg/day empagliflozin for 8 weeks. Magnification of D, sham, E10, and E25: x400.

The histological data of testis in the sham and D group showed abnormal seminiferous tubule epithelium morphology with many vacuoles and epithelial ruptures with respect to the control group (**Figure 2**). They showed atrophic seminiferous tubules, with only 1–3 layers of cells in a disordered arrangement with many vacuoles. The spermatogenic cells were shown sporadic diffused, and a large decrease was observed in the sperm count. Empagliflozin attenuated the pathological changes in the seminiferous tubules of E10 and E25 groups compared to the sham and D groups and preserved the architecture of cells in the testicular tissues in this respect. Furthermore, histological samples of the distal portion of the head of the epididymis in the control animals showed a packed mass of non-motile sperm with high sperm storage (**Figure 3**). Injection of streptozotocin markedly decreased sperm storage in the sham and D groups compared to the control ones. Empagliflozin administration increased sperm storage in the E10 and E25 groups in a dose-dependent manner.

Our data of histology showed that the diameter of seminiferous tubules, the numbers of spermatogonia, spermatocyte, and spermatid (**Table 1**) significantly decreased in animals from the D and sham groups compared with the control group (p ≤ 0.05). In the E25 group, a significant increase was observed in the numbers of spermatogonia, spermatocyte, and spermatid cells in comparison to sham and D groups. STZ treatment had no effects on Sertoli cells.

**Figure 4:**
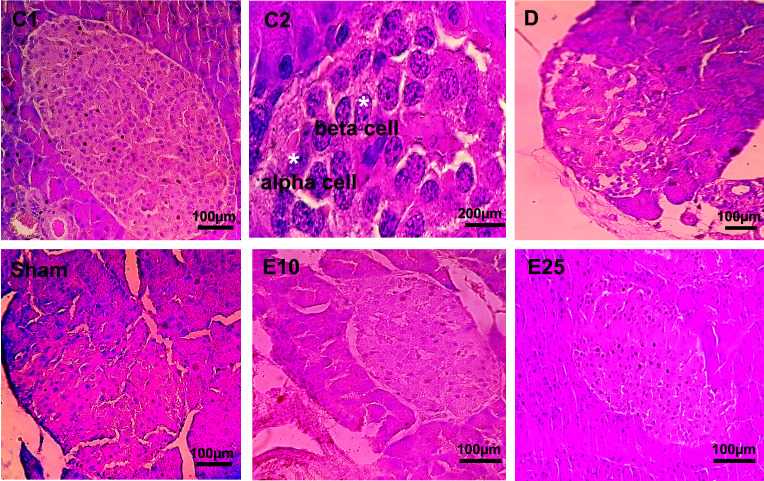
Pancreas cross-section. C1: control rats (magnification x400), C2: control rats (magnification x1000), D: diabetes group, sham: diabetic rats that received distilled water for 8 weeks, E10: empagliflozin-10 group (diabetic rats that received 10 mg/kg/day empagliflozin for 8 weeks, E25: empagliflozin-25 group (diabetic rats that received 25 mg/kg/day empagliflozin for 8 weeks. Magnification of D, sham, E10, and E25: x400.

Similar results were observed in pancreatic tissue samples (**Figure 4**). Control animals showed no noticeable disarrangement. Normal islet cells with a clear boundary, integrated nucleus, and obvious structure without vacuolization were seen in the control tissues. In the sham and D tissues, the morphology of islet cells was irregular with unclear boundaries, a smaller nucleus, and vacuolization in the cytoplasm. E10 and E25 groups showed improvement in this respect in a dose-dependent manner. Injection of STZ significantly decreased the number of islets, the percent of β-cells, and the islet size (**Table 1**) in animals from the D and sham groups compared with the control group (P ≤ 0.05). The E25 group showed improvement in this respect in comparison to the sham and D groups (P ≤ 0.05).

### Gene expression analysis by quantitative real-time PCR

**Figure 5:**
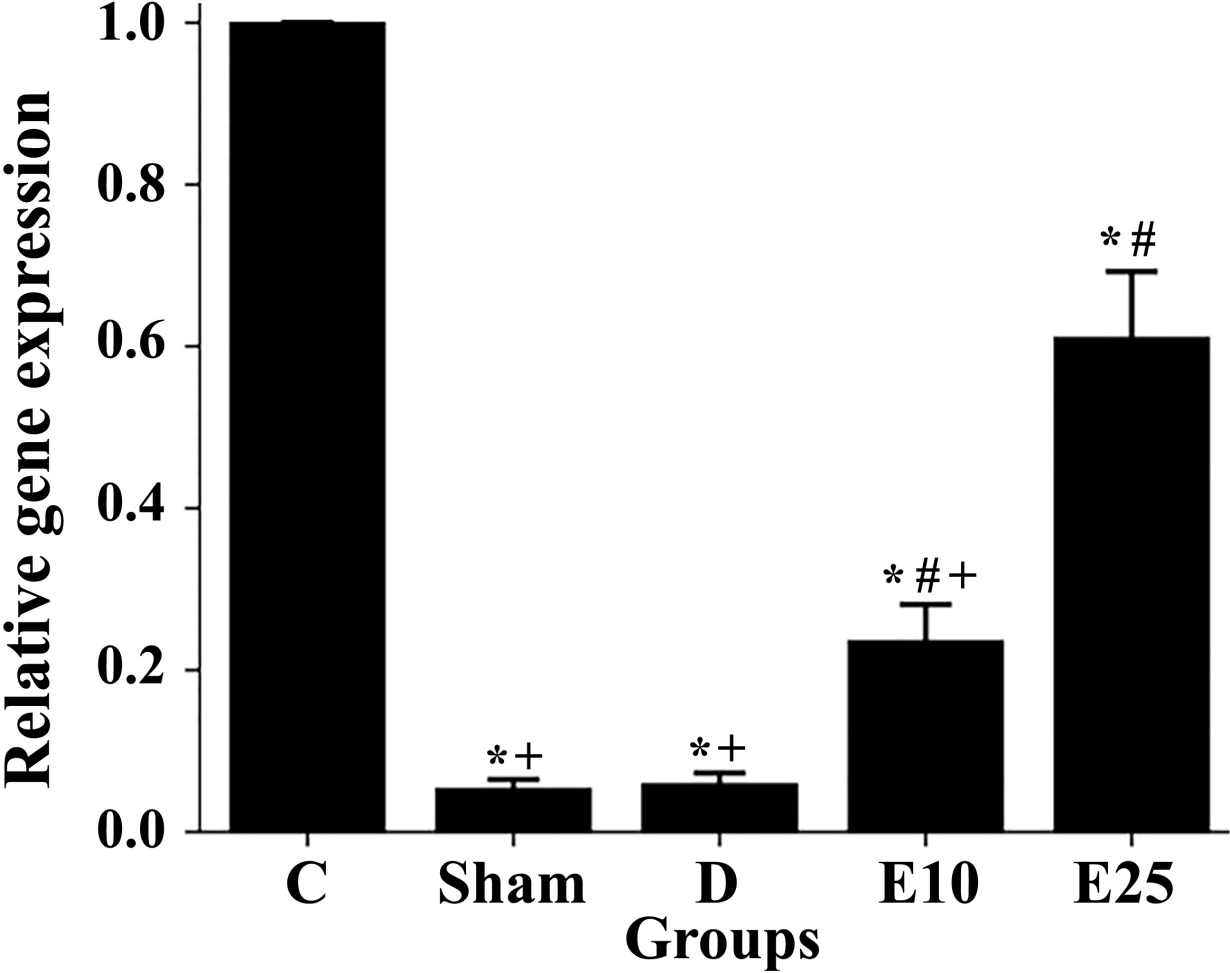
Changes in the kisspeptin mRNA levels in response to the empagliflozin in control and diabetes rats. C: control rats, D: diabetes group, sham: diabetic rats that received distilled water for 8 weeks, E10: empagliflozin-10 group (diabetic rats that received 10 mg/kg/day empagliflozin for 8 weeks, E25: empagliflozin-25 group (diabetic rats that received 25 mg/kg/day empagliflozin for 8 weeks. * Statistically different from the control rats (p ≤ 0.05), # statistically different from the diabetes rats (p ≤ 0.05), + statistically different from the E25 rats (p ≤ 0.05).

The relative expression of the *Kisspeptin* gene was studied using quantitative real-time RT-PCR. The expression of the *kisspeptin* gene significantly decreased in the diabetic and sham rats in comparison to control rats (**Figure 5**). Empagliflozin treatment at the concentration of 10 mg/kg increased the expression of the *kisspeptin* gene in the E10 group when compared to the D and sham groups. Increasing the concentration of empagliflozin to 25 mg/kg caused a further increase in *kisspeptin* expression in the E25 group (p ≤ 0.05). This observation may indicate that empagliflozin affects diabetes features in a dose-dependent manner.

## Discussion

In the present study, empagliflozin improved the expression of the kisspeptin gene and reproduction parameters in STZ-induced diabetic male rats. One of the main health concerns of diabetes is male infertility problems due to a decrease in testosterone and alteration in sperm quality and spermatogenesis. Indeed, glucose metabolism and insulin levels are required for the maintenance of spermatogenesis and fertility ability (15–17). Schoeller et al. (2012) showed that insulin rescued male fertility through the regulation of the function of the hypothalamic-pituitary-gonadal axis and thus regulated the production of FSH, LH, and testosterone hormones in diabetic animals (43). Kisspeptin is a hypothalamic neuropeptide hormone that directly regulates the release of gonadotrophin-releasing hormone (GnRH) which modulates the production of gonadotrophins including luteinizing hormone (LH), follicle-stimulating hormone (FSH)) and downstream sex hormones such as testosterone and oestradiol in both males and females (18– 23). Also, studies in primates, rodents, amphibians, and fish have demonstrated an important function of kisspeptin in germ cell progression, modulation of sperm function, and testicular steroidogenesis (24). Hsu et al. (2014) have shown that KISS1R is expressed in the acrosome region of spermatids, spermatozoa, and Leydig cells (22). In the present study, the injection of STZ reduced the expression of the kisspeptin gene which is in line with the finding of Castellano et al. (2006) (44). Also, it caused severe changes in the reproductive system such as alteration in spermatogenesis, sperm quality, gonadosomatic index, levels of gonadotropins (FSH and LH) and testosterone, and histopathological changes in testis tissue. Diabetes decreased the weight of the testis and caused some pathologic changes in seminiferous tubules such as disarrangement, vacuolization, and a decrease in their diameter. Also, the number of spermatogonia, spermatocyte, spermatid, normal sperms, and motile sperms significantly decreased in treated animals which is in line with the findings of previous studies (15–17, 43, 45–47). Administration of empagliflozin increased the expression of the kisspeptin gene in the E10 and E25 animals in comparison to the D and sham ones. Empagliflozin increased the weight of the testis and improved the number of spermatogonia, spermatocyte, spermatid cells, motile sperms, and abnormal sperms in treated animals, too. The biochemical finding also confirmed the histological data and the improvement of reproductive function in empagliflozin-treated animals. The amount of LH and testosterone hormone was increased in treated animals which confirms the role of kisspeptin in the regulation of GnRH release and production of sex hormones shown in previous studies (18, 23).

This increase in the expression of Kisspeptin may be due to an increase in leptin levels following the administration of empagliflozin. Leptin and adiponectin are hormones with a critical role in glucose homeostasis as well as body fat amount (48, 49). Several studies report that decreased levels of leptin and adiponectin are associated with insulin resistance and the development of diabetes (48–51). In addition, leptin is involved in stimulating kisspeptin expression and increasing the kisspeptin levels in the hypothalamus (52, 53). Therefore, it seems that reproductive problems are caused in STZ-induced diabetic male rats, at least in part, by a decrease in leptin and kisspeptin. In the current study, injection of STZ increased blood glucose, dysregulated lipid profile, decreased the amount of insulin and leptin, and developed diabetes. Histological analysis of pancreas tissue in diabetic animals showed a decrease in β-cells and degradation of Langerhans islets accompany by widespread vacuolization, too.

Also, it has been suggested that impaired adipose tissue function due to the dysregulation of leptin and adiponectin may be involved in the induction of oxidative stress and the development of metabolic disorders. Fruhbeck et al. (2017) have shown that lowering adiponectin and leptin may contribute to the increase of oxidative stress and the development of metabolic disorders (54). In fact, Oxidative stress, inflammation, and obesity which subsequently can lead to the production of oxidative stress and inflammation have a critical role in the development of diabetes (1–7). According to obtained data, the amount of MDA increased in diabetic animals while the levels of SOD and CAT decreased; which indicates the induction of oxidative stress. After the administration of empagliflozin, the amount of MDA decreased in the E10 and E25 animals while the levels of SOD and CAT increased. MDA, SOD, and CAT are known as the principal parameters of oxidative stress. SOD and CAT are enzymes with antioxidant functions and MDA - the final product of lipid peroxidation by ROS- is an oxidative stress marker (55). Evidence indicates that empagliflozin has anti-oxidant and anti-inflammatory activity (29). Therefore, the antioxidant activity of empagliflozin may be due to an increase in leptin. An increase in oxidative stress can affect the reproductive system, too (6, 7, 46, 56). In fact, spermatozoa are more susceptible to oxidative stress due to the high levels of fatty acids in their plasma membranes. Previous studies have shown an increase in sperm membrane cholesterol and an increase in oxidative stress in obese individuals (3, 4, 6, 7).

The expression pattern of kisspeptin indicates that in addition to regulating reproductive function, kisspeptin is also involved in the regulation of energy metabolism and glucose hemostasis. Kisspeptin is expressed in a variety of tissues including the neurons of the hypothalamus, adrenal gland, pancreas, liver, and adipose tissue (19–21). Studies by Izzi-Engbeaya et al. (2018) have shown that kisspeptin could affect B-cell function and increase insulin secretion which is important in understanding the relationship between reproductive function and metabolic condition (52). The amount of insulin improved after the administration of empagliflozin, which may be the result of the increase in the kisspeptin expression. Our data revealed that atrophic changes of islets in pancreatic tissues and disarrangement were less observable in empagliflozin-treated rats. Treatment with empagliflozin improved the number of islets and β-cells and preserved the integrity of Langerhans islets. The benefits of empagliflozin could be due to the decrease in oxidative stress, too. Empagliflozin is a sodium-glucose co-transporter 2 (SGLT2) inhibitor that lower blood sugar levels in an insulin-independent manner by suppressing the reabsorption of glucose in the proximal tubule of kidneys upon inhibition of SGLT2 (28, 29). It increased urinary excretion of glucose in the E10 and E25 animals in comparison to the D and sham ones in a dose-dependent manner. It caused weight loss while stimulating water consumption which confirms the results shown in a previous study by Ferrannini et al. (2015) (57). As mentioned before, there is a critical link between hyperglycemia and oxidative stress (1). Therefore, it may conclude that improvement in oxidative stress in empagliflozin-treated animals, at least in part, by a decrease in glucose levels.

## Conclusion

In summary, our data showed that hyperglycemia, hyperlipidemia, reproductive impairment, increasing oxidative stress, and inhibition of kisspeptin expression occur in streptozotocin-induced diabetes rats. Administration of empagliflozin significantly ameliorated symptoms of diabetes. Based on the results of this study, the application of empagliflozin therapy to diabetic individuals may be beneficial in increasing blood hormones (insulin, LH, and testosterone), lowering oxidative stress, improvement of leptin production, reducing the risk of reproductive complications, and correcting the expression of kisspeptin. It probably has promising antidiabetic potential and may improve the male infertility of diabetic subjects. However, further studies are required to investigate the interaction of empagliflozin with other antidiabetic supplements and to clarify the mechanism responsible for the improvement of reproductive function. Also, it would be better to measure the serum levels of insulin, leptin, and sex hormones weekly or biweekly. Empagliflozin did not have any effects on blood lipid profile and adiponectin levels, so a combination drug that contains an SGLT2 inhibitor and a fat-lowering drug is recommended. To our knowledge, this is the first experimental evidence for the potential impact of empagliflozin on kisspeptin expression in diabetic subjects.

## Data availability statement

The original contributions presented in the study are included in the article/supplementary material. Further inquiries can be directed to the corresponding author.

## Ethics statement

The animal study was reviewed and approved by Animal Care and Use Committee of Islamic Azad University, Science and Research Branch (permit number: IR.IAU.SRB.REC.1399.184)

## Author contributions

PD, NR, PY, ZH: Contributed to conception and design. PD: Contributed to all experimental work, data and statistical analysis, and interpretation of data. NR and PY; Were responsible for overall supervision. ZH; Drafted the manuscript, which was revised by NR and PY. All authors contributed to the article and approved the submitted version.

## Funding

Partial financial support was received from Islamic Azad University, Science and Research Branch, for conducting this study

## Conflict of interest

The authors declare that the research was conducted in the absence of any commercial or financial relationships that could be construed as a potential conflict of interest.

## Publisher’s note

All claims expressed in this article are solely those of the authors and do not necessarily represent those of their affiliated organizations, or those of the publisher, the editors and the reviewers. Any product that may be evaluated in this article, or claim that may be made by its manufacturer, is not guaranteed or endorsed by the publisher.
